# ICTV Virus Taxonomy Profile: *Circoviridae*

**DOI:** 10.1099/jgv.0.000871

**Published:** 2017-08-08

**Authors:** Mya Breitbart, Eric Delwart, Karyna Rosario, Joaquim Segalés, Arvind Varsani, ICTV Report Consortium

**Affiliations:** ^1^​ College of Marine Science, University of South Florida, 140 7th Avenue South, Saint Petersburg, FL 33701, USA; ^2^​ Blood Systems Research Institute, San Francisco, CA 94118, USA; ^3^​ UAB, Centre de Recerca en Sanitat Animal (CReSA, IRTA-UAB), Campus de la Universitat Autònoma de Barcelona, Facultat de Veterinària, UAB, 08193 Bellaterra, Spain; ^4^​ Departament de Sanitat i Anatomia Animals, Facultat de Veterinària, UAB, 08193 Bellaterra, Spain; ^5^​ The Biodesign Center for Fundamental and Applied Microbiomics, Center for Evolution and Medicine, and School of Life Sciences, Arizona State University, Tempe, AZ 85287-5001, USA

**Keywords:** *Circoviridae*, *Circovirus*, *Cyclovirus*, *ICTV report*, taxonomy

## Abstract

The family *Circoviridae* comprises viruses with small, circular, single-stranded DNA (ssDNA) genomes, including the smallest known animal viruses. Members of this family are classified into two genera, *Circovirus* and *Cyclovirus*, which are distinguished by the position of the origin of replication relative to the coding regions and the length of the intergenic regions. Within each genus, the species demarcation threshold is 80 % genome-wide nucleotide sequence identity. This is a summary of the International Committee on Taxonomy of Viruses (ICTV) Report on the taxonomy of the *Circoviridae*, which is available at www.ictv.global/report/circoviridae.

## Abbreviations

ORF, open reading frame; Rep, replication associated protein; Cp, capsid protein; ori, origin of replication; RCR, rolling circle replication.

## Virion

**Fig. 1.**
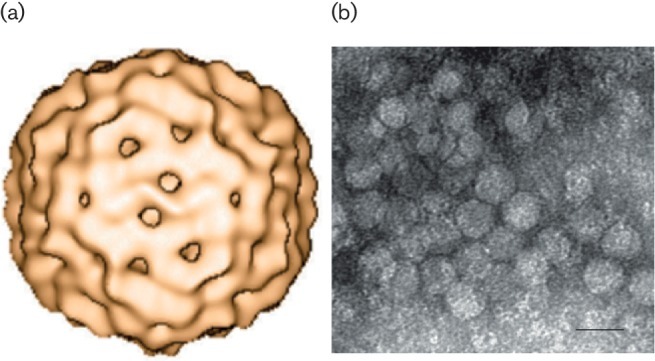
(a) 3D reconstruction of porcine circovirus 2 using cryo-electron microscopy. A structural model comprising 60 subunits (*T*=1) arranged in 12 pentameric morphological units has been proposed [1]. (b) Negative-stained transmission electron micrograph of porcine circovirus 2 (provided by Carolina Rodríguez-Cariño and Joaquim Segalés, CReSA, Spain). Scale bar=20 nm.

Virions, which have only been visualized for a few members of the genus *Circovirus*, are non-enveloped and have an icosahedral *T*=1 symmetry with a diameter of 15–25 nm [1–3] (Table 1, Fig. 1). Members of the genus *Cyclovirus* have only been described through sequence-based analyses and no structural data are available.

**Table 1.** Characteristics of the family *Circoviridae*

Typical member:	porcine circovirus 1 (AF071879), species *Porcine circovirus 1*, genus *Circovirus*
Virion	Non-enveloped, icosahedral *T*=1 symmetry, 15–25 nm diameter
Genome	Monopartite, circular, single-stranded DNA of 1.7–2.1 kb
Replication	Rolling circle replication
Translation	From at least two mRNAs encoding the replication-associated and capsid proteins
Host Range	*Circovirus*: mammals, birds and fish; *Cyclovirus*: unconfirmed for most species
Taxonomy	More than 70 species in the genera *Circovirus* and *Cyclovirus*

## Genome

**Fig. 2.**
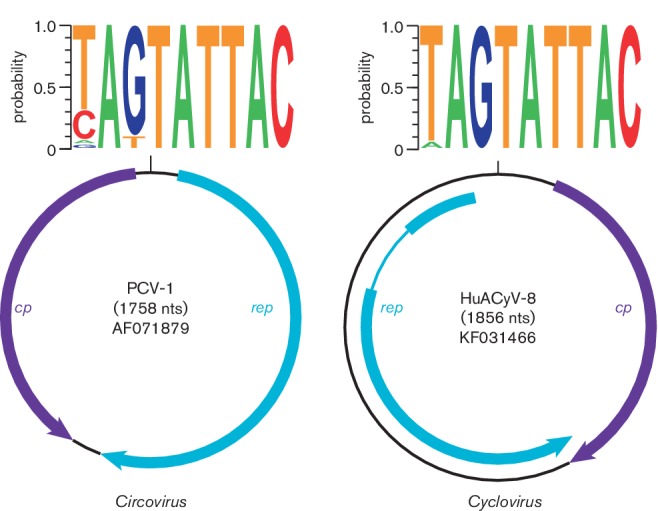
Genome schematics illustrating the major open reading frames (ORFs) characteristic of members of the family *Circoviridae*. Members of the family have two major ORFs encoding replication-associated (Rep) and capsid (Cp) proteins, as well as a conserved nonanucleotide motif marking the origin of replication. The nonanucleotide motif sequence is depicted through sequence probability logos generated in Weblogo 3. The *rep* gene of human-associated cyclovirus 8, a representative of the *Cyclovirus* type species, is interrupted by an intron.

Both genera include viruses with small, covalently closed, circular ssDNA genomes. Their genomes range in size from 1.7 to 2.1 kb and contain two major (>600 nt) open reading frames (ORFs), which encode the replication-associated (Rep) and capsid (Cp) proteins. Members of the genera *Circovirus* and *Cyclovirus* are distinguished by the location of the origin of replication (*ori*) relative to the coding regions, and the length of the intergenic regions (Fig. 2) [4]. Members of the genus *Circovirus* have the *ori* on the same strand as the *rep* ORF, whereas members of the genus *Cyclovirus* have the putative *ori* on the same strand as the *cp* ORF [5]. Circovirus genomes are characterized by two intergenic regions between the major ORFs; however, the intergenic region between the 3′ ends of the major ORFs in cyclovirus genomes is either absent or consistently smaller [6]. In addition, introns have been identified within the ORFs of several cyclovirus genomes, while none have been observed for members of the genus *Circovirus*.

## Replication

The *ori* is characterized by a conserved nonanucleotide motif [(T/n)A(G/t)TATTAC] (Fig. 2) at the apex of a stem–loop structure located between the 5′ ends of Rep- and Cp-encoding ORFs [4, 7]. In characterized members of the genus *Circovirus*, the Rep protein is thought to initiate replication through the rolling circle replication (RCR) mechanism by nicking the virion-sense strand between positions 7 and 8 of the nonanucleotide motif [8]. RCR involves the production of a dsDNA replicative form by host DNA polymerases and the generation of viral ssDNA from the replicative form template. Both circovirus and cyclovirus Rep proteins contain conserved domains that are important for RCR. Putative Rep-binding domains characterized by iterative sequences near the *ori* have been identified for members of both genera [9, 10].

## Taxonomy

The family *Circoviridae* includes two genera, *Circovirus* and *Cyclovirus* [4]. Members of the genus *Circovirus* have only been identified in vertebrates, whereas members of the genus *Cyclovirus* have been identified in both vertebrates and invertebrates [5]. The type species for the genus *Circovirus* is *Porcine circovirus 1* and the type species for the genus *Cyclovirus* is *Human-associated cyclovirus 8*. The species demarcation threshold for viruses of the family *Circoviridae* is 80 % genome-wide nucleotide sequence identity.

## Resources

Full ICTV Online (10th) Report: www.ictv.global/report/circoviridae.

## References

[1] Crowther RA, Berriman JA, Curran WL, Allan GM, Todd D (2003). Comparison of the structures of three
circoviruses: chicken anemia virus, porcine circovirus type 2, and beak and
feather disease virus. J
Virol.

[2] Ritchie BW, Niagro FD, Latimer KS, Lukert PD, Steffens WL 3rd (1990). Ultrastructural, protein composition, and
antigenic comparison of psittacine beak and feather disease virus purified
from four genera of psittacine birds. J Wildl
Dis.

[3] Todd D, Niagro FD, Ritchie BW, Curran W, Allan GM (1991). Comparison of three animal viruses with circular
single-stranded DNA genomes. Arch
Virol.

[4] Rosario K, Breitbart M, Harrach B, Segalés J, Delwart E (2017). Revisiting the taxonomy of the family
*Circoviridae*: establishment of the genus
*Cyclovirus* and removal of the genus
*Gyrovirus*. Arch
Virol.

[5] Rosario K, Duffy S, Breitbart M (2012). A field guide to eukaryotic circular
single-stranded DNA viruses: insights gained from
metagenomics. Arch
Virol.

[6] Li L, Kapoor A, Slikas B, Bamidele OS, Wang C (2010). Multiple diverse circoviruses infect farm animals
and are commonly found in human and chimpanzee
feces. J
Virol.

[7] Mankertz A, Persson F, Mankertz J, Blaess G, Buhk HJ (1997). Mapping and characterization of the origin of DNA
replication of porcine circovirus. J
Virol.

[8] Steinfeldt T, Finsterbusch T, Mankertz A (2006). Demonstration of nicking/joining activity at the
origin of DNA replication associated with the rep and rep' proteins
of porcine circovirus type 1. J
Virol.

[9] Dayaram A, Potter KA, Moline AB, Rosenstein DD, Marinov M (2013). High global diversity of cycloviruses amongst
dragonflies. J Gen
Virol.

[10] Steinfeldt T, Finsterbusch T, Mankertz A (2001). Rep and Rep' protein of porcine circovirus
type 1 bind to the origin of replication *in
vitro*. Virology.

